# Paranormal Belief, Thinking Style and Delusion Formation: A Latent Profile Analysis of Within-Individual Variations in Experience-Based Paranormal Facets

**DOI:** 10.3389/fpsyg.2021.670959

**Published:** 2021-06-28

**Authors:** Kenneth Graham Drinkwater, Neil Dagnall, Andrew Denovan, Christopher Williams

**Affiliations:** Department of Psychology, Manchester Metropolitan University, Manchester, United Kingdom

**Keywords:** paranormal belief/experience, reality testing, emotion-based reasoning, belief in science, delusion proneness, latent profile analysis

## Abstract

This study examined the degree to which within-individual variations in paranormal experience were related to belief in the paranormal, preferential thinking style, and delusion formation. A sample of 956 non-clinical adults completed measures assessing experience-based paranormal indices (i.e., paranormal experience, paranormal practitioner visiting, and paranormal ability), paranormal belief, belief in science, proneness to reality testing deficits, and emotion-based reasoning. Latent profile analysis (LPA) combined the experience-based indices to produce six underlying groups. Inter-class comparison via multivariate analysis of variance (MANOVA) indicated that both breadth and intensity of experiential factors were associated with higher belief in in the paranormal, increased proneness to reality testing deficits, and greater emotion-based reasoning. Belief in science, however, was less susceptible to experiential variations. Further analysis of reality testing subscales revealed that experiential profiles influenced levels of intrapsychic activity in subtle and intricate ways, especially those indexing Auditory and Visual Hallucinations and Delusional Thinking. Collectively, identification of profiles and inter-class comparisons provided a sophisticated understanding of the relative contribution of experiential factors to differences in paranormal belief, belief in science, proneness to reality testing deficits, and emotion-based reasoning.

## Introduction

National surveys report that belief in the paranormal remains widespread within contemporary Western societies (i.e., United Kingdom, Ipsos, 1998, Ipsos, 2003, United States, Gallup: Newport and Strausberg, 2001; Moore, 2005). Indeed, a 2005 Gallup poll (Moore, 2005) observed that three in four Americans acknowledged at least one paranormal belief. Concomitant with belief, the reporting of paranormal experiences is also relatively common (e.g., Schmied-Knittel and Schetsche, 2005; Castro et al., 2014; Dagnall et al., 2016). For instance, Dagnall et al. (2016) noted that 42% of a British university-based sample experienced at least one paranormal occurrence. Studies from other geographical areas have produced comparable findings (e.g., Germany, Schmied-Knittel and Schetsche, 2005; America, McCready and Greeley, 1976; and Latin American, Montanelli and Parra, 2002; Marks, 2021). Consideration of literature reveals also that experiencers frequently report multiple occurrences (Castro et al., 2014; Dagnall et al., 2016).

Irwin et al. (2013) postulate that discernment of a paranormal experience comprises two fundamental processes. Perception of an anomaly (inexplicable stimulus), and subsequent ascription of causation to paranormal entities or powers (Lange et al., 2019). Thus, belief provides an interpretative lens that structures comprehension of anomalous phenomena. Explicitly, it provides a cognitive framework for organising life events, so that they appear intellectually coherent, and experiences represent internal misattributions to external (“paranormal”) forces (Irwin, 1994; Dagnall et al., 2020). Congruent with this notion, several studies report a positive association between belief in and experience of the paranormal (Glicksohn, 1990; Dagnall et al., 2016). Noting the interpretative nature of this process, researchers often describe outcomes as subjective paranormal experiences (SPEs) (see Neppe, 1990; Dagnall et al., 2016; Drinkwater et al., 2020). Similarly, when faced with anomalous phenomena, disbelief can facilitate rejection of paranormal explanations in favour of conventional elucidations (Dagnall et al., 2017a).

This constructionist view accords with sociological and psychological research. Sociologically, the cultural source hypothesis depicts paranormal experiences as products of tradition, or imaginary happenings created, and shaped by social custom (Hufford, 1982; McClenon, 1994). At an individual cognitive level, this aligns with the psychological concept of worldview, the idea that overarching expectations and assumptions (beliefs) about the world shape understanding of reality and existence (Overton, 1991; Miller and West, 1993; Koltko-Rivera, 2004; Dagnall et al., 2015b). In this context, direct (i.e., personal encounters) and indirect experience (second-hand accounts, academic knowledge, media, etc.) provide confirmatory evidence for beliefs.

Personal life events can also promote supernatural credence (Glicksohn, 1990). This viewpoint is embodied within experiential source theory (Hufford, 1982), which advocates that a significant portion of traditional supernatural belief is associated with accurate observations, interpreted rationally. Explicitly, that certain types of anomalous experience provide a source of recurring beliefs (Hufford, 1982, 2005; Bennett, 1987; McClenon, 1994). Consistent with experiential source theory, the exchange and evaluation of information about anomalous occurrences, in part, contributes to the creation of folk religions. An example of this is the Night or Old Hag tradition, where reference to a communal, paranormal entity is used to explain elements of sleep paralysis, such as feeling immobilised by a malevolent presence (Hufford, 2005). Other studies provide affirmative evidence for the experiential source hypothesis. Pertinent to the present paper, these reference the observation of “common elements” in reported cases of spontaneous extrasensory perception, precognitive dreams, apparitional experiences, and contact with the dead (see Rhine, 1981; Emmons, 1982; McClenon, 1990). Similarly, in support of the experiential source theory, several authors note that individuals frequently cite personal paranormal encounters as the motivation for belief(s) (McClenon, 1982, 2000; Blackmore, 1984; Irwin, 1991).

In rational terms, since individuals can explain anomalous stimuli via a range of non-supernatural elucidations, experiential factors are less likely to generate paranormal attributions than beliefs. Acknowledging this, the tendency to ascribe paranormality to experience is heightened under specific conditions. For example, when a personal event creates uncertainty and/or anxiety, the psychological desire for understanding and control, can truncate objective decision-making and encourage an overreliance on self-generated (internal/subjective) data (Frenkel-Brunswik, 1949, 1951). This includes the endorsement of alternative, scientifically unsubstantiated (paranormal) beliefs (see Williams and Irwin, 1991; Houran and Williams, 1998; Hart et al., 2013). Moreover, experience and belief can function in an interactive, reciprocal manner so that experiences stimulate interest and belief in the supernatural (van Elk, 2017), and beliefs encourage the search for confirmatory personal paranormal occurrences (Dagnall et al., 2015a; Drinkwater et al., 2020). This dynamic synergy is an inherent feature of Van Leeuwen and van Elk's (2019) Interactive Religious Experience Model (IREM). Within the IREM, general belief causes individuals to seek situations that activate corresponding experiences. In the case of religion, these often take the form of low-level agency-intuitions (i.e., feelings of presence). These are important since they reflect and influence belief formation.

In terms of previous research, the observed co-occurrence of experience(s) and beliefs supports the notion that the constructs are often connected within individuals. Indeed, studies generally report a moderate-positive correlation (Cohen, 1992) (e.g., Glicksohn, 1990; Musch and Ehrenberg, 2002; Dagnall et al., 2016). Regarding the observed relationship, there are important points to note. Firstly, variations in association strength arise from the employment of different measurement instruments. There is no commonly agreed index of experience, and researchers have historically assessed belief using a variety of scales, which encompass diverse but related content (i.e., dimensions) (see Dagnall et al., 2010a). Secondly, studies using the Australian Sheep Goat Scale (ASGS; Thalbourne and Delin, 1993), a widely employed index of belief, require cautious interpretation as the scale conflates belief, experience, and ability. Particularly, items sample all three constructs (e.g., “I believe I have had personal experience of ESP”). In cases where experiences overlap with elements present within the ASGS this likely results in correlation inflation (i.e., ESP, psychokinesis, and life after death, see Drinkwater et al., 2018b).

Thirdly, since the RPBS assesses a wide breadth of construct content (i.e., Psi, Witchcraft, Superstition, Spiritualism, Extraordinary Life Forms, and Precognition) that varies in frequency of perceived occurrence and plausibility, the scale may not accurately assess the general relationship between paranormal belief and experience. This notion is supported by Dagnall et al. (2016), who observed considerable variation in experience(s) as a function of type. For instance, extrasensory perception was commonly reported, whereas psychokinesis was rarely acknowledged. There is also an important conceptual misalignment between haunting encounters and RPBS dimensions. The nearest indirect correspondences are the Traditional Religious Belief (i.e., soul, Devil, God, and heaven and a hell) and Spiritualism (i.e., astral projection, out-of-body experiences, reincarnation, and communicate with the dead) subscales. Hence, although haunt-related experiences are relatively commonly reported (i.e., ghosts, poltergeists, and apparitions) there is no direct referent within the RPBS.

Thirdly, the reported relationship between belief and experience is more compelling when Gignac and Szodorai's (2016) normative guidelines for interpreting correlation effect sizes are applied (i.e., relatively large, *r* > 0.39). Finally, the two constructs are not mutually inclusive. Hence, not all believers have had a corresponding experience, nor are all experiencers high in paranormal belief (Drinkwater et al., 2017a; Lange et al., 2019; Wahbeh et al., 2019). Indeed, individuals can label paranormal events as “supernatural” without any great faith or conviction (Drinkwater et al., 2013, 2017a). Thus, SPEs represent just one ontological facet that informs or reinforces belief. The relatively small proportion of variance shared by the two constructs demonstrates the relative independence of experience and belief. Moreover, experience and belief are differentially related to psychological variables (Rattet and Bursik, 2001). Collectively, these factors help to explain why research on the experiential basis of belief has generated inconsistent findings (Castro et al., 2014).

### Limitations of Previous Work

Noting these issues, it is apposite to conclude that due to methodological limitations, previous work examining the relationship between paranormal experience and belief has provided useful, but restricted insights. The major limitation being overreliance on a narrow index of person-centred paranormal experience, direct reported encounters (i.e., SPEs). This focus ignores the fact that involvement in the paranormal takes many forms, such as actively engaging with practitioners (i.e., Mediums, Psychics, Fortune-Tellers, and Spiritualists), and normative influences (e.g., family and peers) (Hill et al., 2018; Drinkwater et al., 2019).

Additionally, there are potentially important differences between people who report single vs. multiple instances, and those that believe they possess psychic abilities. Consistent with this supposition, Zingrone et al. (2009) observed positive relationships between aura viewers (i.e., perceiving lights, glimmers, or force fields around the human body) and reports of other psychic experiences (extrasensory perception, apparitions, and out-of-body experiences). This suggests that multiple experiencers have increased openness to “other” paranormal encounters and perhaps in some instances self-perceived abilities. Consequently, they may differ from individuals who experience one off phenomenon, or just one type of paranormal experience. Furthermore, it is possible to conceptualise experiences as predominately dispositional (internally generated by an inherent, personal quality, e.g., precognition) vs. situational (externally created by context/situation, e.g., ghost sighting). From this perspective, perceived possession of abilities infers differences in type and frequency of reported paranormal experience(s).

Considerations such as these suggest that the measurement of specific phenomena (i.e., personal encounters) alone is unlikely to offer full insights into the effects of paranormal experience on individual beliefs and cognitions. The importance of including an assortment of experiential facets is not a new idea as demonstrated by the Anomalous Experiences Inventory (AEI; Gallagher et al., 1994). The AEI contains subscales measuring experiences, abilities, fear, and beliefs. The AEI, however, is rarely used in contemporary research since the Revised Paranormal Belief Scale (RPBS; Tobacyk, 2004) and Australian Sheep Goat Scale (ASGS; Thalbourne and Delin, 1993) have become the mostly widely used measures of belief (Drinkwater et al., 2017b).

Another limitation derives from researchers' use of variable-based analytical methods, such as path models and regression. For example, Dagnall et al. (2016) employed correlation analyses when assessing indices of paranormal experience. A variable-centred approach anticipates that findings are an estimate of associations among discrete variables averaged across the whole population, correspondingly assumed to be homogeneous (Orri et al., 2017). This variable-centred approach is problematic because it fails to examine how indices of experience relate within individuals and ignores the observation that they interrelate (interact) in complex ways.

### Addressing Limitations

Noting these limitations, the present paper used a range of experience-based indices (i.e., paranormal experience, paranormal practitioner visiting, and paranormal ability). From a methodological perspective, the approach of including and amalgamating multiple measures was advantageous since it sampled greater construct breadth and acknowledged intra-respondent (person-centred) variations within participants. Latent profile analysis (LPA) was used to combine experience-based indices. LPA categorises individuals into configural profiles based on varying degrees of probabilities (for a review of LPA see Spurk et al., 2020).

The emergent composite measure, like the AEI, included direct experience and self-professed ability. Regarding the AEI dimension of Fear, this was replaced by paranormal practitioner visiting. This decision was informed by the rationale that visiting reflected an “active” desire to seek out paranormal experience, whereas Fear was likely to promote “avoidance.” These factors together encompassed, theoretically important experiential aspects. It was necessary to develop new measures because preceding research has typically employed limited measures of paranormal experience (e.g., SPE), which focus on restricted aspects of direct encounters.

In this context, the use of LPA to combine person-centred factors represented an important conceptual development. Particularly, the use of profiles recognised the heterogeneous nature of individual paranormal experience and facilitated the identification of subtle differences between category members. Previous work using LPA, has provided useful insights into how interactions between belief in the paranormal and schizotypy are related to differential performance on probabilistic reasoning tasks (Denovan et al., 2018). Similarly, research using cluster analysis has contributed greatly to academic understanding of paranormal experience and belief. Clustering algorithms partition data into subsets based on similarity or dissimilarity (Frades and Matthiesen, 2010). For instance, Goulding (2004, 2005) used cluster analysis to investigate how relationships between schizotypal factors were associated with belief in the paranormal.

### The Present Paper

Observing the potential of LPA, the current paper examined the degree to which profile membership was associated with differences in paranormal belief and cognitive-perceptual information processing; specifically, preferential thinking style (i.e., intuitive vs. experiential) and factors related to delusion formation in general populations (i.e., reality testing and emotion-based reasoning). This builds upon the body of academic work that has investigated the extent to which preferential thinking style predicts endorsement of scientifically unsubstantiated beliefs and mediates related cognitive-perceptual processes (i.e., schizotypy) (Dagnall et al., 2017a; Barron et al., 2018; Denovan et al., 2020). Although the relationship between belief in the paranormal and intuitive thinking is well-established, relatively few studies have examined whether this applies also to experiences (Irwin and Wilson, 2013). Those that have, posit a similar positive association between paranormal experiences and intuitive-experiential processing (Wolfradt and Watzke, 1999; Wolfradt et al., 1999; Irwin and Wilson, 2013).

Researchers often adopt a dual processing approach to examine differences in preferential thinking style. This refers to the notion that two independent, but parallel operating systems influence decision-making (Epstein et al., 1996). Although dual processing conceptualisations vary across models, theorists generally agree on the importance of the distinction between automatic (implicit) and controlled (explicit) processes (see Shirzadifard et al., 2018). Consistent with this perspective, preceding research has drawn heavily on the intuitive-experiential vs. analytical-rational dichotomy encapsulated within Cognitive-Experiential Self-Theory (CEST; Epstein, 1983, 1990).

CEST advocates that experiential processing is automatic, rapid, unconscious, and holistic, whereas rational processing is deliberate, slow, and conscious. Correspondingly, experiential processing draws on feelings and emotional experiences (subjective, internal mental activity) (Epstein et al., 1996), while rational processing seeks to validate meaning by referring to impartial evidence (objective, external data) (Epstein, 1994). Although these processes contribute jointly to reasoning and operate in parallel, one system typically preponderates.

Consistent with earlier work, indirect, proxy measures assessed differences in thinking style (e.g., Dagnall et al., 2017a; Denovan et al., 2017; Barron et al., 2018). Congruently, the reality testing sub-scale of the Inventory of Personality Organisation (IPO-RT; Lenzenweger et al., 2001) assessed intuitive-experiential thinking, and the Belief in Science Scale (BISS; Farias et al., 2013) indexed rational-analytical thinking. The IPO-RT assesses the ability to differentiate self from non-self, intrapsychic from external stimuli, and the capacity to maintain empathy with ordinary social criteria of reality (Kernberg, 1996). This conceptualisation is consistent with the information processing approach to belief generation proposed by Langdon and Coltheart (2000).

This model explains delusions in terms of impairments within the cognitive belief system. Delusions with ordinary content, arise because of an extreme (but normal) attentional bias. Precisely, the failure to critically evaluate hypotheses based on misperceptions and misinterpretations of ambiguous first-person experience. In the case of bizarre delusions, two deficits occur. Firstly, damage to sensory and/or attentional-orienting mechanisms creates an aberrant perception. Secondly, there is a failure in belief evaluation. This explains why bizarre delusions both contain unusual content and are implausible. The implausibility arises from an inability to suspend the natural tendency to favour direct first-person evidence. This bias prevents objective critical evaluation of information.

Accordingly, researchers use the IPO-RT to measure inclination to engage in intuitive-experiential processing (Dagnall et al., 2010a; Denovan et al., 2017), proneness to reality testing deficits (Irwin, 2004; Dagnall et al., 2017a), and delusional thinking (Irwin et al., 2012; Dagnall et al., 2017b). This reflects intersection between these constructs. Explicitly, the delineation of reality testing as the ability to assess the validity of beliefs and suppositions via reference to external data sources. The application to delusions derives from the supposition that delusion formation within non-clinical populations is associated with proneness to reality testing deficits and emotion-based reasoning (EBR) (i.e., the extent which judgements are based on affective responses) (Irwin et al., 2012; Dagnall et al., 2017a; Drinkwater et al., 2021).

From this viewpoint, proneness to reality testing deficits is the failure to rigorously assess and continuously evaluate information, and EBR denotes preference for emotional (vs. logical) data. These psychological constructs align closely with the contemporary definition of delusion(s) (Drinkwater et al., 2021). This no longer references falsity (Dagnall et al., 2017b), but instead views delusions as beliefs founded on insufficient scrutiny of evidence. These are persistently held in the face of conflicting evidence and are accepted for their emotional appeal than for logical coherence (American Psychiatric Association, 2013). Although, emotional significance in paranormal beliefs is typically lower than with psychotic delusions (Cella et al., 2012), research with delusional patients has confirmed that EBR generally plays an important role in delusion formations and maintenance (e.g., Beck et al., 2008).

The shift in emphasis from falsity was beneficial to the conceptual understanding of belief in the paranormal because, unlike baseless psychotic delusions, it is not possible to definitively disprove the existence of supernatural phenomena. Thus, the inclusion of inadequate critical evaluation and emotional emphasis as defining cognitive processes legitimises the generalisation of findings from clinically defined delusions, to paranormal beliefs in non-clinical populations. This concurs with the clinically informed notions of delusions as beliefs arising from faulty interpretation of anomalous experiences (Garety and Freeman, 1999) and/or inadequate evidence (Irwin, 2009; Coltheart et al., 2010; Irwin et al., 2012). From this perspective, paranormal beliefs within general populations represent non-psychotic delusions (Irwin et al., 2012).

In support of this supposition, EBR and the tendency to suspend reality testing predicted scores on the two dimensions of the Survey of Anomalous Experiences (SAE) (Irwin et al., 2013). The SAE measures proneness to anomalous experiences (the tendency to experience anomalous or uncanny experiences) and proneness to paranormal attributions (the degree to which respondents ascribe experiences to specific paranormal process). In addition to this, EBR and proneness to reality testing deficits predicted intuitive–experiential thinking style and were not associated with rational thinking (Irwin and Wilson, 2013). These results concurred with studies that found EBR was a prognosticator paranormal belief intensity (e.g., Irwin et al., 2012).

These findings concur with the postulation that individuals endorse paranormal beliefs based on emotional appeal, and that this process is the foundation for enduring beliefs (Irwin et al., 2012). This outcome aligned with Sappington (1990), who found that potential to explain personal experiences heighten emotion-based reasoning, and increased judgment of phenomena as paranormal. These findings applied to experience, suggest that scores on belief in the paranormal, proneness to reality testing deficits, belief in science and EBR will vary as a function of profile membership.

In conclusion, LPA examined the degree to which within-individual variations in experience were related to belief in the paranormal, preferential thinking style, and delusion formation. Congruent with preceding research, it was predicted that profiles with greater levels of paranormal experience would score higher on belief in the paranormal and the measures of intuitive thinking/delusion formation (i.e., IPO-RT and EBR), and lower on critical thinking (i.e., BISS).

## Methods

### Design

To identify heterogeneous, experience-based latent profiles a cross-sectional design was used. Profiles comprised paranormal experience, paranormal practitioner visiting, and paranormal ability. Analysis then examined relationships between emergent profiles and belief in the paranormal, preferential thinking style, and delusion formation.

### Respondents

The sample comprised 956 respondents, (Mean age, *M*) = 33.02 years, *SD* = 14.64, range 18–83. There were 342 males (36%), *M* = 37.16 years, *SD* = 15.51, range 18–82; and 614 females (64%), *M* = 30.71 years, *SD* = 13.61, range 18–83. For all variables, skewness and kurtosis values were within the recommended range of−2.0 to +2.0 (Byrne, 2010). Respondent recruitment was via Bilendi, an online multi-channel management platform for data collection. Bilendi is an established provider of good quality representative samples (see Häusermann et al., 2020; Salak et al., 2021).

The researchers requested a sample of UK-based respondents aged 18 years and over. This was the only exclusion criteria. Data accessed via respondent recruitment panels are generally more diverse and far reaching than traditional student samples. These advantages are not detrimental to quality and are commensurate with traditional samples in terms of demographics and responses to established surveys (Kees et al., 2017; Miller et al., 2017).

### Measures

#### Experiential Paranormal Factors

The survey contained a section assessing experiential paranormal factors. This was subdivided into paranormal experiences, paranormal practitioner visiting, and perceived ability. These measures were adapted from (see Dagnall et al., 2016; Drinkwater et al., 2018a, 2021).

#### Paranormal Experience

Items asked respondents whether they had experienced a range of psychic phenomena. Presented experiences were associated with psi and life after death, and included communication with the dead, psychic occurrence, mediumship, spiritualism, telepathy, precognition, premonition, and remote viewing. These items indexed frequently reported paranormal experiences (see Dagnall et al., 2016), and represent core receptive elements of belief in the paranormal (Drinkwater et al., 2018b).

Each psychic phenomenon was accompanied by a clear delineation. The use of definitions ensured that participants were responding to conceptual classifications, rather than personal interpretations. For instance, “Precognition is paranormal awareness (knowing) that an event in the future will occur. In the context of this definition, have you ever personally experienced precognition?” Summation of experiences produced a total ranging from 0 (no experience) to 8 (experienced all phenomena). A frequency scale followed each experience item (EXP) (“On how many occasions? Once, Between 2 and 5, or More than 5”). This approach to the measurement of self-reported paranormal experiences is well-established (see Dagnall et al., 2016; Drinkwater et al., 2020, 2021). These scales were dichotomous and demonstrated a good level of reliability according to the Kuder-Richardson-20 (KR-20) coefficient, EXP KR-20 = 0.799; FREQ KR-20 = 0.839.

#### Paranormal Practitioner Visiting

A further item set, using a dichotomous format (yes vs. no), asked respondents whether they had visited paranormal practitioners associated with psi and life after death. Designated categories centred on main industries (i.e., Mediums, Psychics, Spiritualists, and Fortune-Tellers). The reliability for this measure was satisfactory, KR-20 = 0.713. For each category, if respondents provided an affirmative response, a further item assessed frequency of visits (either once, between 2 and 5, or more than 5). Reliability for this was good, KR-20 = 0.849. A final item asked how accurate (in percentage terms, 0-100) was the information provided. Alpha and omega reliability for this continuous scale was good, α = 0.824; ω = 0.826.

#### Perceived Personal Ability

This section asked respondents about their perceived personal abilities (i.e., mediumship, psychic, spiritualism, and fortune-telling); e.g., “To what extent (in percentage terms, 0-100) do you believe that you possess mediumship abilities?”. Internal consistency for this was excellent, α = 0.912; ω = 0.917. A final item asked respondent whether they were paranormal practitioners (yes vs. no).

#### Belief in the Paranormal

The Manchester Metropolitan University New (MMU-N) scale assessed belief in the paranormal. This measure has featured in published studies (Dagnall et al., 2014, 2020; Drinkwater et al., 2021) and provides total and dimensional scores (hauntings, superstition, religious belief, alien visitation, ESP, PK, astrology, and witchcraft) (Dagnall et al., 2010a,b,c). The MMU-N comprises 50-items presented as statements (e.g., “Some people have visions of the future, which come true”). Participants respond to these using a seven-point Likert scale (ranging from 1, strongly disagree, to 7, strongly agree). Overall, the MMU-N is conceptually coherent and psychometrically robust. Particularly, the scale and subfactors possess good face validity and excellent internal reliability (Dagnall et al., 2010a). Furthermore, the measure has demonstrated good concurrent validity (Dagnall et al., 2014). In the present report, excellent alpha and omega reliability existed, α = 0.963; ω = 0.965.

#### Reality Testing

The IPO-RT contains 20-items presented as statements (e.g., “I have heard or seen things when there is no apparent reason for it”). Respondents indicate agreement on a five-point Likert scale, with responses ranging from 1 = never true, to 5 = always true. Totalling items produces scores between 20 and 100. Higher scores are indicative of proneness to reality testing deficits and greater reliance on intrapsychic activity (intuitive-experiential thinking) (Dagnall et al., 2018). The IPO-RT has demonstrated good internal and external reliability, and construct validity (Lenzenweger et al., 2001; Dagnall et al., 2018).

The IPO-RT is a superior measure of intuitive-experiential processing because it assesses extensive construct content. This includes cognitive, perceptual, emotional, and social components of internal and self-orientation (Dagnall et al., 2018). Indeed, Dagnall et al. (2017a) supported this notion via verifying the existence of four distinct but related subfactors. Auditory, and visual hallucinations, delusional thinking (possessing beliefs contrary to reality), social deficits (difficulties reading social cues) and confusion (inability to understand feelings and sensations). Other widely used measures, such as the Faith in Intuition subscale of the Rational-Experiential Inventory (REI; Epstein et al., 1996), focus only on the role of feelings, instincts, and emotions in decision-making (Pennycook et al., 2016). Therefore, the IPO-RT has become an established index of individual inclination to engage in intuitive-experiential processing (subjective thinking). In this study, excellent reliability emerged for the overall measure, α = 0.925; ω = 0.926. The subfactors of auditory and visual hallucinations (α = 0.848; ω = 0.850), delusional thinking (α = 0.858; ω = 0.860), social deficits (α = 0.755; ω = 0.763), and confusion (α = 0.682; ω = 0.691) demonstrated good to acceptable internal consistency.

#### Belief in Science

The Belief in Science Scale (BISS; Farias et al., 2013) assesses the degree to which respondents endorse the virtues of science. The measure is composed of 10 statements (e.g., “Science is the most efficient means of attaining truth”). Respondents indicate their level of agreement via a six-point Likert scale (1 = Strongly Disagree, to 6 = Strongly Agree). Researchers can produce total scores by summing items (10 to 60) and can average these to produce scores ranging from 1.0 to 6.0. The BISS has high internal consistency and validity (Farias et al., 2013). The measure also has demonstrated invariance (i.e., gender, form, factor structure), and item intercepts for the unidimensional structure (Dagnall et al., 2019). In the current study, internal reliability was excellent, α = 0.920; ω = 0.922.

#### Emotion-Based Reasoning

The Emotion-Based Reasoning (EBR) subscale of the Cognitive Biases Questionnaire (Peters et al., 2014) is a 6-item measure of the degree to which decision-making is based upon affective reactions. Items are framed in the context of a short vignette and respondents select one of three available options that best describes their feelings about the outlined situation. Each item has a three-point scale (1 = absence of bias; 2 = presence of bias with some qualification; and 3 = presence of bias). EBR is computed as a total across the items, hence scores range from 6 to 18. The Cognitive Biases Questionnaire possesses good psychometric properties (Cronbach alpha, α = 0.89; test-retest reliability, *r* = 0.92) (Peters et al., 2014). In the current research, alpha was acceptable (α = 0.630; ω = 0.639). (see Taber, 2018). This result aligned with previous research (e.g., Drinkwater et al., 2018b), additionally the subscale possessed a satisfactory mean inter-item correlation of 0.227.

## Procedure

Respondents accessed materials via a web-link. Prior to item presentation, potential respondents received background information about the research project. This outlined the nature of the investigation and details about ethics. To progress, it was necessary for respondents to indicate informed consent. Respondents then provided basic demographic information (i.e., age and preferred gender) before receiving the measures. Within these, guidelines asked respondents to carefully read and answer all questions, take their time, and respond in an open and honest manner. Items were organised into sections: paranormal experience and abilities, MMU-N, BISS, IPO-RT, and EBR. To prevent order effects section presentation rotated across respondents. Respondents worked through the items at their own pace until they reached the end of survey, at which point they received the debrief.

As aforementioned, this study used a cross-sectional design, data collection occurred at one point in time. A frequent criticism of this method is that it is prone to common method variance (CMV) (Spector, 2019). To remedy this the researchers employed procedural devices (Krishnaveni and Deepa, 2013). Explicitly, section instructions created psychological distance between scales by accentuating differences between constructs and measures (Podsakoff et al., 2003). Furthermore, instructions reduced the potential for evaluation apprehension and social desirability effects by telling respondents that there were no right or wrong responses, and that they should answer questions honestly.

## Ethics Statement

Ethical approval was granted for a series of studies examining psychological and neuropsychological factors associated with self-professed psychic ability/mediumship by the Manchester Metropolitan University Faculty of Health, Psychology and Social Care Ethics Committee (October 2018).

## Analysis

Aside from latent profile analysis (LPA), which required Mplus version 7 (Muthén and Muthén, 2012), analyses utilised SPSS 26. Preliminary analysis examined descriptive statistics. Subsequently, exploratory LPA established latent group affiliation based on indices of paranormal experiences and abilities (Paranormal Experience, Paranormal Practitioner Visiting, and Paranormal Ability). Assessment of model fit involved evaluating a 1-class model, followed by examining models with an increasing quantity of latent profiles until the inclusion of additional profiles was not justified.

Consultation of the following indices determined the optimal quantity of latent profiles: the Akaike Information Criterion (AIC; Akaike, 1987), the Bayesian Information Criterion (BIC; Schwarz, 1978), the sample-size adjusted BIC (ssaBIC; Sclove, 1987), the Lo-Mendell-Rubin-adjusted likelihood ratio test (LMR-A-LRT; Lo et al., 2001), and a standardised measure of entropy (Ramaswamy et al., 1993). Lower values suggest superior fit for AIC, BIC, and ssaBIC. The LMR-A-LRT includes a *p*-value and determines statistical significance (or otherwise) in fit. Entropy values reflect the classification quality of participants, with values above 0.8 indicative of a sound separation of identified profiles relative to the data (Ramaswamy et al., 1993).

Resultant latent profiles represented a group variable (independent variable) for assessing differences in relation to Paranormal Belief, Reality Testing, Belief in Science, and Emotion-based Reasoning. Further analysis investigated differences on IPO-RT (Reality Testing) subscales.

## Results

### Descriptive Statistics

Prior to analysis, data screening removed outliers. Four z-scores marginally greater than 3.25 were transformed to the next highest score (Tabachnick and Fidell, 2001). As Table 1 indicates, indices of paranormal experiences and abilities (Paranormal Experience, Paranormal Practitioner Visiting, and Paranormal Ability) were positively intercorrelated. Additionally, Belief in the Paranormal, Proneness to Reality Testing Deficits, and Emotion-Based Reasoning correlated positively, whereas Belief in Science was negatively associated with paranormal experiences and abilities. Although, several of the relationships were significant, they were in the small range (*r* = 0.10 to 0.30) (see Cohen, 1992). These represent more meaningful associations when interpreted using the guidelines of Gignac and Szodorai (2016) (i.e., small, *r* = 0.11; medium, *r* = 0.19, and large, *r* = 0.29).

**Table 1:** Descriptive statistics and intercorrelations among all study variables.

**Variable**	***Mean***	***SD***	**1**	**2**	**3**	**4**	**5**	**6**	**7**	**8**	**9**	**10**	**11**
1. Paranormal Experience				0.55^**^	0.41^**^	0.48^**^	0.40^**^	0.24^**^	−0.18^**^	0.38^**^	0.41^**^	0.24^**^	0.18^**^
2. Paranormal Practitioner Visiting					0.26^**^	0.34^**^	0.20^**^	0.13^**^	−0.10^*^	0.19^**^	0.21^**^	0.13^**^	0.07^*^
3. Paranormal Ability						0.44^**^	0.45^**^	0.30^**^	−0.15^**^	0.40^**^	0.45^**^	0.35^**^	0.19^**^
4. Paranormal Belief	174.48	54.85					0.52^**^	0.37^**^	−0.34^**^	0.49^**^	0.52^**^	0.30^**^	0.33^**^
5. Reality Testing	42.46	14.22						0.51^**^	−0.13^**^	0.91^**^	0.93^**^	0.81^**^	0.65^**^
6. Emotion-based Reasoning	8.39	2.23							−0.23^**^	0.44^**^	0.51^**^	0.42^**^	0.26^**^
7. Belief in Science	38.82	11.37								−0.14^**^	−0.15^**^	−0.06	−0.03
8. Auditory and Visual Hallucinations	13.23	5.00									0.76^**^	0.64^**^	0.63^**^
9. Delusional Thinking	13.99	5.82										0.71^**^	0.48^**^
10. Social Deficits	7.61	3.22											0.40^**^
11. Confusion	8.41	2.65											

* *indicates p < 0.05;*

** *indicates p < 0.001*.

### Latent Profile Analysis

For LPA model comparisons see Table 2. Initial assessment of 1-class and 2-class models was undertaken. AIC, BIC, and ssaBIC indices revealed the superior fit of the 2-class model. This was supported by the LMR-A-LRT, which indicated significant improvement over the 1-class model. Evaluation of 2-class and 3-class solutions found that the 3-class solution was superior, due to lower AIC, BIC, ssaBIC statistics, higher entropy (0.958 vs. 0.946), and a significant LMR-A-LRT *p*-value.

**Table 2:** Fit of competing latent profile models.

**Model**	**AIC**	**BIC**	**ssaBIC**	**LMR-A**	**LMR-A *p-*value**	**Entropy**
1-class	10202.221	10231.403	10212.346			
2-class	9180.811	9229.439	9197.679	693.230	<0.001	0.946
3-class	8748.389	8816.468	8772.004	424.942	0.008	0.958
4-class	8335.268	8422.798	8365.631	406.319	<0.001	0.975
5-class	7952.878	8079.309	7996.734	251.743	<0.001	0.978
6-class	7452.631	7559.612	7489.740	151.327	<0.001	0.981
7-class	7593.278	7739.160	7643.881	−306.919	0.770	0.989

*AIC, Akaike Information Criterion; BIC, Bayesian Information Criterion; ssaBIC, sample-size adjusted BIC; LMR-A, Lo-Mendell-Rubin-adjusted likelihood ratio test*.

Next, the 4-class solution demonstrated superior fit to the 3-class solution; lower AIC, BIC, ssaBIC statistics, higher entropy (0.975 vs. 0.958), and a significant LMR-A-LRT *p*-value. Iteratively, the 5-class model indicated a significant improvement over the 4-class solution, possessing lower AIC, BIC, ssaBIC statistics, higher entropy (0.978 vs. 0.975), and a significant LMR-A-LRT *p*-value. Subsequent analysis of a 6-class model suggested superiority to the 5-class model, with lower AIC, BIC, ssaBIC statistics, higher entropy (0.981 vs. 0.978), and a significant LMR-A-LRT *p*. Finally, the 7-class solution indicated no significant improvement over the 6-class model; hence, the optimal solution was identified and there was no further consideration of solutions.

**Figure 1:**
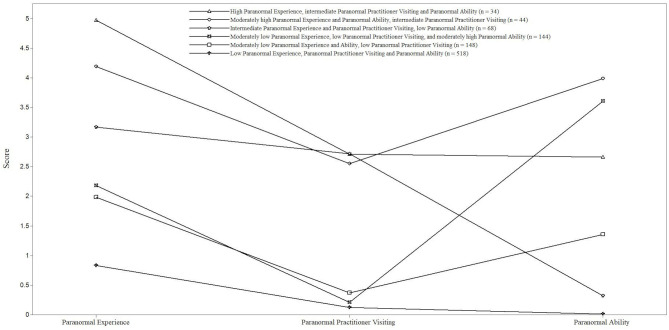
Pattern of mean scores for Paranormal Experience, Paranormal Practitioner Visiting, and Paranormal Ability as a function of latent profile.

Figure 1 provides a visual representation of relative scores on Paranormal Experience, Paranormal Practitioner Visiting, and Paranormal Ability. Table 3 displays the profiles organised in sequence from higher to lower overall scores.

**Table 3:** Profiles organised in sequence from higher to lower overall scores.

**Grouping**	**Percentage of sample**	**Description**
Class 1	3.6% (*n* = 34)	High Paranormal Experience, intermediate Paranormal Practitioner Visiting and Paranormal Ability
Class 2	4.6% (*n* = 44)	Moderately high Paranormal Experience and Paranormal Ability, intermediate Paranormal Practitioner Visiting
Class 3	7.1% (*n* = 68)	Intermediate Paranormal Experience and Paranormal Practitioner Visiting, low Paranormal Ability
Class 4	15.0% (*n* = 144)	Moderately low Paranormal Experience, low Paranormal Practitioner Visiting, and moderately high Paranormal Ability
Class 5	15.5% (*n* = 148)	Moderately low Paranormal Experience and Ability, low Paranormal Practitioner Visiting
Class 6	54.2% (*n* = 518)	Low Paranormal Experience, Paranormal Practitioner Visiting and Paranormal Ability

Average latent class probabilities for most likely latent class membership was 0.972 for class 1, 0.997 for class 2, 0.971 for class 3, 0.997 for class 4, 0.966 for class 5, and 0.999 for class 6, indicating good overall discrimination.

### Association of Latent Profiles With Paranormal Belief, Reality Testing, Belief in Science, and Emotion-Based Reasoning

Multivariate analysis of variance (MANOVA) examined the effect of latent profile membership on the following outcome variables: Belief in the Paranormal, Belief in Science, Proneness to Reality Deficits, and Emotional-Based Reasoning (see Table 4). Analysis revealed a significant main effect of group, Pillai's trace = 0.316, *F*_(20, 3800)_ = 16.314, *p* < 0.001, η^2^ = 0.079 (medium effect size). Significant effects for group were observed in relation to all outcome variables.

**Table 4:** The effects of group (latent profile) in relation to Paranormal Belief, Reality Testing, Belief in Science, and Emotion-based Reasoning.

	**Dependent variable**						
	**Paranormal belief**	**Reality testing**	**Belief in science**	**Emotion-based reasoning**			
	**ANOVA**					**MANOVA**	
	***F ^***df***^* (*Sig*.; **η^2^**)**	***F ^***df***^* (*Sig*.; **η^2^**)**	***F ^***df***^* (*Sig*.; **η^2^**)**	***F ^***df***^* (*Sig*.; **η^2^**)**	**Pillai's trace**	***F ^***df***^* (*Sig*.)**	****η^2^****
Variable							
Group	62.50 ^5, 950^ (<0.001; 0.24)	2.89 ^5, 950^ (<0.001; 0.19)	79.04 ^5, 950^ (<0.001; 0.03)	53.01 ^5, 950^ (<0.001; 0.09)	0.31	16.31 ^20, 3800^ (<0.001)	0.07
	Pairwise comparisons (mean differences) between classes						
Class contrast	Mean diff. (*Sig*.)	Mean diff. (*Sig*.)	Mean diff. (*Sig*.)	Mean diff. (*Sig*.)			
Class 1 vs. Class 2	5.26 (1.00)	0.67 (1.00)	−2.49 (1.00)	−0.35 (1.00)			
Class 1 vs. Class 3	30.20 (0.034)	9.72 (0.004)	−4.14 (1.00)	0.65 (1.00)			
Class 1 vs. Class 4	22.15 (0.209)	0.31 (1.00)	−2.63 (1.00)	−0.44 (1.00)			
Class 1 vs. Class 5	44.09 (<0.001)	7.32 (0.035)	−4.94 (0.285)	0.29 (1.00)			
Class 1 vs. Class 6	78.49 (<0.001)	15.59 (<0.001)	−6.97 (0.006)	1.31 (0.006)			
Class 2 vs. Class 3	24.93 (0.107)	9.05 (0.004)	−1.65 (1.00)	1.00 (0.220)			
Class 2 vs. Class 4	16.88 (0.629)	−0.36 (1.00)	−0.14 (1.00)	−0.09 (1.00)			
Class 2 vs. Class 5	38.82 (<0.001)	6.64 (0.040)	−2.45 (1.00)	0.64 (1.00)			
Class 2 vs. Class 6	73.22 (<0.001)	14.91 (<0.001)	−4.48 (0.179)	1.67 (<0.001)			
Class 3 vs. Class 4	−8.04 (1.00)	−9.41 (<0.001)	1.50 (1.00)	−1.09 (0.006)			
Class 3 vs. Class 5	13.89 (0.662)	2.40 (1.00)	−0.79 (1.00)	0.35 (1.00)			
Class 3 vs. Class 6	48.29 (<0.001)	5.86 (0.005)	−2.83 (0.716)	0.66 (0.222)			
Class 4 vs. Class 5	21.93 (0.001)	7.01 (<0.001)	−2.30 (1.00)	0.73 (0.043)			
Class 4 vs. Class 6	56.33 (<0.001)	15.28 (<0.001)	−4.33 (0.001)	1.76 (<0.001)			
Class 5 vs. Class 6	34.40 (<0.001)	8.27 (<0.001)	−2.03 (0.747)	1.02 (<0.001)			

*Class 1 = High Paranormal Experience, intermediate Paranormal Practitioner Visiting and Paranormal Ability (n = 34). Class 2 = Moderately high Paranormal Experience and Paranormal Ability, intermediate Paranormal Practitioner Visiting (n = 44). Class 3 = Intermediate Paranormal Experience and Paranormal Practitioner Visiting, low Paranormal Ability (n = 68). Class 4 = Moderately low Paranormal Experience, low Paranormal Practitioner Visiting, and moderately high Paranormal Ability (n = 144). Class 5 = Moderately low Paranormal Experience and Ability, low Paranormal Practitioner Visiting (n = 148). Class 6 = Low Paranormal Experience, Paranormal Practitioner Visiting and Paranormal Ability (n = 518)*.

*Post-hoc* pairwise comparisons with Bonferroni correction (Table 4) revealed that the **class 1** group (High Paranormal Experience, intermediate Paranormal Practitioner Visiting and Paranormal Ability) demonstrated high Belief in the Paranormal, Proneness to Reality Testing, and lowest Belief in Science. Correspondingly, **class 6** (Low Paranormal Experience, Paranormal Practitioner Visiting and Paranormal Ability) reported considerably lower levels of Belief in the Paranormal, Proneness to Reality Testing and Emotion-based reasoning, and higher Belief in Science. Across remaining profiles (**classes** 2, 3, 4, and 5), scores were moderate to intermediate. An exception to this trend was Emotion-based reasoning, where **class 2** (Moderately high Paranormal Experience and Paranormal Ability, intermediate Paranormal Practitioner Visiting), and class 4 (Moderately low Paranormal Experience, low Paranormal Practitioner Visiting, and moderately high Paranormal Ability) scored highest, though not in both instances significantly different from class 1 (High Paranormal Experience, intermediate Paranormal Practitioner Visiting and Paranormal Ability).

### IPO-RT Subscale Analysis

Noting overall effects for IPO-RT, additional analysis using MANOVA investigated subscales differences (Auditory and Visual Hallucinations, Delusional Thinking, Social Deficits, and Confusion) (Table 5). Analysis revealed a significant main effect of group, Pillai's trace = 0.242, *F*_(20, 3800)_ = 12.252, *p* < 0.001, η^2^ = 0.061 (medium effect size). Significant group effects were also observed. *Post-hoc* pairwise comparisons with Bonferroni correction (Table 4) indicated that **class 6** (Low Paranormal Experience, Paranormal Practitioner Visiting and Paranormal Ability) scored significantly lower on Auditory and Visual Hallucinations, Delusional Thinking, and Social Deficits. The subfactor of Confusion was an exception to this trend.

**Table 5:** The effects of group (latent profile) in relation to Reality Testing subfactors.

	**Dependent variable**						
	**Auditory and visual hallucinations**	**Delusional thinking**	**Social deficits**	**Confusion**			
	**ANOVA**					**MANOVA**	
	***F ^***df***^* (*Sig*.; **η^2^**)**	***F ^***df***^* (*Sig*.; **η^2^**)**	***F ^***df***^* (*Sig*.; **η^2^**)**	***F ^df^* (*Sig*.; η^2^)**	**Pillai's trace**	***F ^***df***^* (*Sig*.)**	**η^2^**
Variable							
Group	38.72 ^5, 950^ (<0.001; 0.17)	48.12 ^5, 950^ (<0.001; 0.20)	23.69 ^5, 950^ (<0.001; 0.11)	7.44 ^5, 950^ (<0.001; 0.04)	0.24	12.25 ^20, 3800^ (<0.001)	0.06
	Pairwise comparisons (mean differences) between classes						
Class contrast	Mean diff. (*Sig*.)	Mean diff. (*Sig*.)	Mean diff. (*Sig*.)	Mean diff. (*Sig*.)			
Class 1 vs. Class 2	1.31 (1.00)	−0.61 (1.00)	−0.11 (1.00)	0.07 (1.00)			
Class 1 vs. Class 3	3.44 (0.004)	4.10 (0.002)	1.54 (1.00)	0.54 (1.00)			
Class 1 vs. Class 4	0.59 (1.00)	−0.08 (1.00)	−2.63 (0.221)	−0.26 (1.00)			
Class 1 vs. Class 5	2.83 (0.015)	2.62 (0.111)	1.30 (0.338)	−0.05 (1.00)			
Class 1 vs. Class 6	5.59 (<0.001)	6.15 (<0.001)	2.37 (<0.001)	0.95 (0.555)			
Class 2 vs. Class 3	2.12 (0.251)	4.71 (<0.001)	1.65 (0.078)	0.46 (1.00)			
Class 2 vs. Class 4	−0.72 (1.00)	0.52 (1.00)	−0.27 (1.00)	−0.34 (1.00)			
Class 2 vs. Class 5	1.51 (0.827)	3.23 (0.005)	1.42 (0.107)	−0.13 (1.00)			
Class 2 vs. Class 6	4.27 (<0.001)	6.76 (<0.001)	2.49 (<0.001)	0.87 (0.524)			
Class 3 vs. Class 4	−2.85 (<0.001)	−4.18 (<0.001)	1.92 (<0.001)	−0.80 (0.501)			
Class 3 vs. Class 5	−0.60 (1.00)	−1.47 (0.752)	−0.23 (1.00)	−0.60 (1.00)			
Class 3 vs. Class 6	2.15 (0.003)	2.05 (0.031)	0.83 (0.476)	0.40 (1.00)			
Class 4 vs. Class 5	2.24 (<0.001)	2.70 (<0.001)	1.69 (<0.001)	0.20 (1.00)			
Class 4 vs. Class 6	5.00 (<0.001)	6.23 (<0.001)	2.76 (<0.001)	1.21 (<0.001)			
Class 5 vs. Class 6	2.76 (<0.001)	3.53 (<0.001)	1.07 (0.002)	1.01 (<0.001)			

*Class 1 = High Paranormal Experience, intermediate Paranormal Practitioner Visiting and Paranormal Ability (n = 34). Class 2 = Moderately high Paranormal Experience and Paranormal Ability, intermediate Paranormal Practitioner Visiting (n = 44). Class 3 = Intermediate Paranormal Experience and Paranormal Practitioner Visiting, low Paranormal Ability (n = 68). Class 4 = Moderately low Paranormal Experience, low Paranormal Practitioner Visiting, and moderately high Paranormal Ability (n = 144). Class 5 = Moderately low Paranormal Experience and Ability, low Paranormal Practitioner Visiting (n = 148). Class 6 = Low Paranormal Experience, Paranormal Practitioner Visiting and Paranormal Ability (n = 518)*.

**Class 1** (High Paranormal Experience, intermediate Paranormal Practitioner Visiting and Paranormal Ability) reported significantly higher Auditory and Visual Hallucination scores in comparison with all classes/groups but **class 2** (Moderately high Paranormal Experience and Paranormal Ability, intermediate Paranormal Practitioner Visiting), and **class 4** (Moderately low Paranormal Experience, low Paranormal Practitioner Visiting, and moderately high Paranormal Ability). In contrast with the general reality testing factor the highest scores across the remaining subfactors occurred for **class 2** (Moderately high Paranormal Experience and Paranormal Ability, intermediate Paranormal Practitioner Visiting) and **class 4** Moderately low Paranormal Experience, low Paranormal Practitioner Visiting, and moderately high Paranormal Ability) (Social Deficits and Confusion).

## Discussion

Latent profile analysis (LPA) identified discrete classes that categorised important variations in paranormal experience and ability. These represented common differentiations in the frequencies of Paranormal Experience, Paranormal Practitioner Visiting, and Paranormal Ability. Accordingly, each profile grouped individuals based on mutually exclusive relationships between experiential indices. For instance, not all experiencers visited paranormal practitioners, nor did they profess supernatural ability. In addition, individuals reported multiple experiences and visited paranormal practitioners, but claimed little or no paranormal ability. Thus, classes provided a nuanced categorisation of sample subpopulations based on heterogeneous paranormal histories. This approach was theoretically important because it acknowledged that people accrue experience in quantitatively and qualitatively different ways. An additional advantage of LPA was the ability to compare emergent classes on levels of paranormal belief and measures of thinking style.

Consistent with this notion that paranormal experience has a broad and varied basis, zero-order correlations indicated that Paranormal Experience, Paranormal Practitioner Visiting, and Paranormal Ability were related but relatively distinct indices. Specifically, shared variance was relatively low: Experience and Ability, 16%; Visiting and Ability, 19%; and Experience and Visiting, 30%. This explains why, when the experiential indices were combined, they produced a discrete series of complex interactions. Acknowledging the phenomenological sophistication of paranormal experiences is an important academic contribution to the area because it extends the conceptualisation of experience. Traditionally, research has focused exclusively on the paranormal experience index, which is defined as the personal ascription of supernatural powers or forces to direct observations or conscious occurrences. These characteristics are inherent within the standard research operationalisation of subjective paranormal experiences (SPEs), as the willingness to attribute supernatural causation to an event or occurrence (Glicksohn, 1990). The fact that SPEs index only this single experiential aspect explains, in part, why they share only a small proportion of variance with belief in the paranormal. This study demonstrated that supernatural credence is informed, shaped, and reinforced by myriad life events beyond the restricted remit of SPEs.

The finding that Paranormal Experience and Belief in the Paranormal shared only 23% common variance illustrates this. Other studies using a variety of equivalent belief measures have reported similar figures (see Dagnall et al., 2016; Drinkwater et al., 2020). Despite providing only a limited assessment of paranormal experience, it is important to recognise that the study of SPEs has historically acted as a useful indicator of individual proclivity to interpret personal events as supernatural (Schouten, 1983, 1986; Neppe, 1984; Persinger and Valliant, 1985; Palmer and Neppe, 2004; Schmied-Knittel and Schetsche, 2005; Simmonds-Moore, 2016). Therefore, findings recommend the continued use of SPEs in combination with other “additive” indices to produce a broader measure of experience. In this context, an important research development is the utilisation of profiles that classify a range of heterogeneous paranormal phenomena.

Another related limitation of previous research is the use of simplified measures. For example, Dagnall et al. (2016) used dichotomies (i.e., experience vs. non-experience), and categorical distinctions (i.e., single vs. multiple experiences 2-5 vs. more than 5). Relative to class profiles, these provide only snapshots of phenomenological influences. Furthermore, the use of these measures as distinct indices ignores the observation that they interrelate in complex ways. In the current paper, the effects of Paranormal Experience were qualified by Paranormal Practitioner Visiting and Paranormal Ability. This was evidenced by differences between class profiles on observed measures.

Due to the number of comparisons, subsequent discussion focuses on the main trends. **Class 5** (Moderately low Paranormal Experience and Ability, low Paranormal Practitioner Visiting) scored higher than **class 6** (Low Paranormal Experience, Paranormal Practitioner Visiting and Paranormal Ability) on Belief in the Paranormal, Proneness to Reality Testing Deficits, and Emotion-Based Reasoning; there was no difference for Belief in Science. This outcome suggests that even moderate levels of intra class variation can heighten scores. Similarly, **class 4** (Moderately low Paranormal Experience, low Paranormal Practitioner Visiting, and moderately high Paranormal Ability) scored higher than **class 5** on Belief in the Paranormal, Proneness to Reality Testing Deficits, and Emotion-Based Reasoning.

Other comparisons revealed also noteworthy outcomes. **Class 4** scored higher Proneness to Reality Testing Deficits and Emotion-Based Reasoning than **class 3** (Intermediate Paranormal Experience and Paranormal Practitioner Visiting, low Paranormal Ability), and **class 2** (Moderately high Paranormal Experience and Paranormal Ability, intermediate Paranormal Practitioner Visiting) demonstrated higher scores on Proneness to Reality Testing Deficits than **class 3**. Finally, no differences were observed between the two highest intensity experience groups: **Class 1** (High Paranormal Experience, intermediate Paranormal Practitioner Visiting and Paranormal Ability) vs. **class 2**. Collectively, results established that profile membership subtly influenced scores on observed variables.

Focusing on outcome measures (see Table 4), significant differences were typically observed for Proneness to Reality Testing Deficits (11 out of 15 comparisons) and Belief in the Paranormal (9 out of 15 comparisons). Significant differences were observed also for Emotion-Based Reasoning (6 out of 15 comparisons). In contrast, Belief in Science comparisons revealed only two significant differences. These findings suggest that variations in the composition of paranormal experience profiles are most likely to manifest as differences in Proneness to Reality Testing deficits and Belief in the Paranormal. The observed differences in Emotion-Based Reasoning, tentatively indicated increased levels of affective driven decision-making in experience groups (**classes 5, 4, 2**, and **1**) relative to the low paranormal experience group (**class 6**). However, this trend for Emotion-Based Reasoning was less pronounced and therefore requires cautious interpretation.

Collectively, findings supported the notion that greater breadth and intensity of experiential factors were associated with higher Belief in the Paranormal, Proneness to Reality Testing Deficits and Emotion-Based Reasoning. In contrast, Belief in Science was less sensitive to experiential variations; only small differences were observed between profiles.

The effect of profile membership on Proneness to Reality Testing Deficits was demonstrated further by inter class comparisons of IPO-RT subscales. These revealed that **class 6** (Low Paranormal Experience, Paranormal Practitioner Visiting and Paranormal Ability) scored significantly lower on Auditory and Visual Hallucinations, Delusional Thinking, and Social Deficits than the other classes. Additionally, **class 1** (High Paranormal Experience, intermediate Paranormal Practitioner Visiting and Paranormal Ability), **class 2** (Moderately high Paranormal Experience and Paranormal Ability, intermediate Paranormal Practitioner Visiting), and **class 4** (Moderately low Paranormal Experience, low Paranormal Practitioner Visiting, and moderately high Paranormal Ability) scored relatively higher than **class 3** (Intermediate Paranormal Experience and Paranormal Practitioner Visiting, low Paranormal Ability) and **class 5** (Moderately low Paranormal Experience and Ability, low Paranormal Practitioner Visiting). Most differences occurred for Auditory and Visual Hallucinations and Delusional Thinking. Overall, IPO-RT subscale comparisons showed that experiential profiles influenced levels of intrapsychic activity in subtle and intricate ways, especially Auditory and Visual Hallucinations and Delusional Thinking.

With reference to previous research, findings accord with the typically reported positive relationship between experience in and belief of the paranormal (Glicksohn, 1990; Musch and Ehrenberg, 2002; Dagnall et al., 2016). Additionally, results indicated that the two constructs interact in intricate ways. Particularly, via a combination of constructionist (Irwin et al., 2013), cultural (Hufford, 1982; McClenon, 1994), and existential factors (Bennett, 1987) that vary within individuals. Thus, profile differences were consistent with the interpretation that experiences stimulate interest and belief in the supernatural (van Elk, 2017), and that beliefs reciprocally encourage the search for confirmatory personal paranormal occurrences (Dagnall et al., 2015a, 2020). Psychologically, this perspective aligns with the concept of worldview, precisely the notion that experiences inform and are comprehended within an overarching cognitive framework that makes the world intellectually coherent and meaningful (Overton, 1991; Miller and West, 1993; Koltko-Rivera, 2004; Dagnall et al., 2015b). Although, it is important to note that experiential factors are not necessarily antecedent to belief. This dynamic synergy is encapsulated within Van Leeuwen and van Elk's (2019) Interactive Religious Experience Model and generalises well to the paranormal experience-belief relationship.

Results were consistent also with preceding work on thinking styles to the extent that profiles with greater levels of paranormal experience and ability tended to score higher on Proneness to Reality Testing Deficits. Explicitly, the notion that attribution of paranormality is associated with intuitive-experiential processing (Wolfradt and Watzke, 1999; Wolfradt et al., 1999; Irwin and Wilson, 2013). Although, this supposition derives from the use of the IPO-RT, which is an indirect measure, the conclusion appears sound since the IPO-RT has become an established index of intuitive-experiential processing/thinking (Dagnall et al., 2017a, 2019; Denovan et al., 2017, 2020).

Rational-analytical processing, in the form of Belief in Science, did not differ consistently as a function of profile membership. While, this finding concurred with Irwin and Wilson's (2013) conclusion that rational thinking was not a significant predictor of either proneness to anomalous experiences or paranormal attributions, it requires qualification. Principally because few studies to date have employed BISS as an index of rational thinking. This means that the effectiveness of BISS to assess critical, evaluative processing remains largely unattested. Indeed, Dagnall et al. (2019) found that only BISS scores above the median (second quartile) produced a reduction in experiential-based thinking. This suggests that only higher levels of belief in science are associated with rational processing. Explicitly, that elevated belief in science reflects greater comprehension of its strengths (and limitations). This tentative conclusion is supported by the observation that a significant difference was observed between class 1 (high paranormal experiential factors) vs. class 6 (low paranormal experiential factors).

Noting this issue, future research should employ a range of critical thinking measures to determine which best predicts paranormal experience. Researchers have previously used this approach to clarify conflicting findings in the domain of paranormal belief and reasoning (Dagnall et al., 2007). Additionally, investigators could utilise direct, objective measures of rational thinking (see Pennycook et al., 2012). Clearly, additional work is required in this area, especially as rational processing appears differentially related to paranormal experience and belief (Irwin and Wilson, 2013), and it remains unclear whether belief in science is an ineffective index of critical thinking.

The observation that higher Emotion-Based Reasoning was concomitant with increased levels of Belief in the Paranormal and Proneness to Reality Testing Deficits, offers tentative support for the notion that paranormal attributions represent a form of non-psychotic delusions within general populations. Explicitly, that attribution of paranormal experiences and abilities derives from persistent ideas based on emotional appeal, which persevere without empirical support and maintain despite the existence of conflicting evidence (Irwin et al., 2012). However, it is important to note that there were subtle variations across class comparisons that suggest that generally emotion-based reasoning is a contributing, rather than defining factor.

Lastly, to advance understanding about the processes associated with belief, subsequent studies could focus on preselecting different types of paranormal experience/belief groupings to examine how they differ on cognitive-perceptual factors associated with endorsement of scientifically unsubstantiated beliefs (e.g., schizotypy, proneness to hallucinations, and delusional ideation). A further elaboration related to variations in paranormal experience/belief could involve investigating how interactions influence health-related factors such as life satisfaction, mental well-being, and psychological outlook.

### Limitations

Outcomes further illustrated the usefulness of mixture models (i.e., profile analyses/latent class) in social sciences (McLachlan and Peel, 2000). Particularly, that they reveal discrete populations of interest as a function of responses to sets of items (Whittaker and Miller, 2020). Regarding the combined experiential indices within this study, the sample comprised several groups. Nevertheless, it is important to note that classes formed via LPA possess relative differences that may not necessarily represent intrinsic meaning or clinical importance (Achterhof et al., 2019).

The authors are aware, despite criticising previous work for using limited indices of paranormal experience, that the measures used in the present study were also relatively restricted in scope and breadth. Explicitly, this paper focused on psychic phenomena and considers only the influence of Paranormal Experience, Paranormal Practitioner Visiting and Perceived Paranormal Ability. Despite being a core element of parapsychology, psychic phenomena reference only a restricted range of subject matter. Other commonly studied facets are religious belief, witchcraft, superstition, psychokinesis, ghosts and haunting, near death experiences, and out of body experiences (see Dagnall et al., 2010a).

Though, there is overlap between psychic phenomena, via the concepts of spiritualism and life after death, other facets such as haunting/ghosts are associated with relatively commonly reported experiences and high levels of endorsement. Hence, future research needs to establish whether similar experiential profiles exist for other important paranormal phenomena. Intuition suggests that the different contextual nature of experiences and their inherent plausibility is likely to produce divergent classes. For example, given that ghost activity is typically context-based and draws less on perceptions of ability, it is likely that emergent classes would differ to those produce for psychic powers.

Regarding extent, while the present study used a wider range of experiential indices than typical studies, the phenomena selected failed to cover the full array of paranormal happenings. Thus, subsequent studies should examine the contribution of additional factors to determine how these influence classes and more generally best predict related factors (i.e., belief in the paranormal). These could include life history (e.g., indirect experiences via accounts of others), social identity (e.g., mixing with experiencers in contexts such as clubs and societies), and involvement with paranormal media (e.g., watching paranormal films and programmes). Although, the outlined “experiences” are indirect as opposed to the direct observations used in this article, they still represent important experiential factors that influence an individual's beliefs and processing style preferences.

A concern with LPA is that recoding of continuous data to produce categorical variables (classes) may result in information loss (Lanza and Rhoades, 2013). Furthermore, LPA can generate profiles that are statistically sound but conceptually ill defined. This was less of a concern in the present paper since the approach was exploratory. Accordingly, the emergent profiles were of theoretical significance because they established the multifaceted, heterogeneric nature of paranormal experience. Explicitly, they indicated that experiential factors combine to effect beliefs and thinking style in intricate and subtle ways. The authors were aware of the descriptive nature of the emergent classes and that additional work is required. Hence, researchers should focus on the improvement of extant indices and the addition of supplementary factors (e.g., family and peer influences). This iterative process is essential for class development and will facilitate profile operationalisation.

A higher number of females compared to males were recruited. Although, this imbalance was commensurate with related research (e.g., Hergovich and Arendasy, 2005; Drinkwater et al., 2012) it potentially limits the generalisability of findings because some studies have previously reported gender differences in paranormal belief (Dag, 1999, Irwin, 1993). Therefore, future research should explore with gender influences profile membership and composition.

Finally, it is important to acknowledge that LPA categories reflect heterogeneity across model dimensions, not classifications of individuals present within the population (Lanza and Rhoades, 2013). Thus, misspecification can occur in the form of identifying too few or too many classes. To limit this, ensuing research could use cross-validation methods, such as double cross-validation (Collins et al., 1994) or progressive elaboration (see Donovan and Chung, 2015). These approaches objectively assess model fit and help to establish class stability. In the case of cross-validation, this does however only provide the best approximation to the true model (Collins et al., 1994).

## Data Availability Statement

The raw data supporting the conclusions of this article will be made available by the authors, without undue reservation.

## Ethics Statement

The studies involving human participants were reviewed and approved by Manchester Metropolitan University Faculty of Health, Psychology and Social Care Ethics Committee (October 2018). Written informed consent for participation was not required for this study in accordance with the national legislation and the institutional requirements.

## Author Contributions

KD focused theoretically, collected the data, analysed the data, developed the article, and reviewed the draft. ND and AD focused theoretically, analysed the data, and developed the article. CW reviewed the draft. All authors contributed to the article and approved the submitted version.

## Conflict of Interest

The authors declare that the research was conducted in the absence of any commercial or financial relationships that could be construed as a potential conflict of interest.
